# Immune Cell Plasticity Allows for Resetting of Phenotype From Effector to Regulator With Combined Inhibition of Notch/eIF5A Pathways

**DOI:** 10.3389/fcell.2021.777805

**Published:** 2021-11-22

**Authors:** Shahnawaz Imam, Pervaiz Dar, Saba Wasim Aziz, Zeeshan A. Zahid, Haider Sarwar, Tamanna Karim, Sarah Faisal, Ibrahim Haseeb, Ahmed S. Naqvi, Rayyan Shah, Amna Haque, Nancy Salim, Juan C. Jaume

**Affiliations:** ^1^Division of Endocrinology, Diabetes and Metabolism, Department of Medicine, College of Medicine and Life Sciences, University of Toledo, Toledo, OH, United States; ^2^Center for Diabetes and Endocrine Research (CeDER), University of Toledo, Toledo, OH, United States; ^3^Faculty of Veterinary Sciences and Animal Husbandry, Sher-e-Kashmir University of Agricultural Sciences and Technology of Kashmir (SKUAST-K), Srinagar, India; ^4^Department of Internal Medicine, Division of Endocrinology, James H. Quillen College of Medicine, East Tennessee State University, Johnson City, TN, United States; ^5^Windsor University School of Medicine, Cayon, West Indies; ^6^College of Art and Sciences, Case Western Reserve University, Cleveland, OH, United States; ^7^Department of Biological Sciences, University of Toledo, Toledo, OH, United States; ^8^Ottawa Hills High School, Ottawa, OH, United States; ^9^Sylvania Northview High School, Toledo, OH, United States; ^10^Austin College, Sherman, TX, United States

**Keywords:** immune modulation, T1D, Treg, Th1/Th17 plasticity, immune reset

## Abstract

Type 1 diabetes (T1D) results from the destruction of pancreatic β-cells caused by an altered immune balance in the pancreatic microenvironment. In humans as well as in mouse models, T cells are well recognized as key orchestrators of T1D, which is characterized by T helper (Th) 1 and Th17 cell bias and/or low/defective T-regulatory cells (Treg), and culminates in cytotoxic T-cell (CTL)-mediated destruction of β-cells. Refitting of immune cells toward the non-inflammatory phenotype in the pancreas may represent a way to prevent/treat T1D. Recently we developed a unique spontaneous humanized mouse model of type 1 diabetes, wherein mouse MHC-II molecules were replaced by human DQ8, and β-cells were made to express human glutamic acid decarboxylase (GAD) 65 auto-antigen. The mice spontaneously developed T1D resembling the human disease. Humanized T1D mice showed hyperglycemic (250–300 mg/dl) symptoms by the 4th week of life. The diabetogenic T cells (CD4, CD8) present in our model are GAD65 antigen-specific in nature. Intermolecular antigen spreading recorded during 3rd–6th week of age is like that observed in the human preclinical period of T1D. In this paper, we tested our hypothesis in our spontaneous humanized T1D mouse model. We targeted two cell-signaling pathways and their inhibitions: eIF5A pathway inhibition influences T helper cell dynamics toward the non-inflammatory phenotype and Notch signaling inhibition enrich Tregs and targets auto-reactive CTLs, rescues the pancreatic islet structure, and increases the functionality of β-cells in terms of insulin production. We report that inhibition of (eIF5A + Notch) signaling mediates suppression of diabetogenic T cells by inducing plasticity in CD4 + T cells co-expressing IL-17 and IFNγ (IL-17 + IFNγ +) toward the Treg cells phenotype.

## Introduction

Treg cells constitute 5–10% of total peripheral T cells in mice as well as in humans. CD4 + Tregs have a role in maintaining immune homeostasis and preventing autoimmune reactivity (Seddiki et al., 2006; Vaeth et al., 2019). Treg cells also regulate other effector T cells functions. The majority of Treg cells are generated in the medullary region of the thymus gland as single positive CD4 T (CD4-SP) cells. Medullary thymocytes expressing higher affinity interactions with different transgene-encoded antigens are required for the development of Treg cells while lower affinity TCR do not have the ability to differentiate into Treg cells (Jordan et al., 2001).

For proper development and function of Treg cells, Tregs are crucially depend on the forkhead box transcription factor FOXP3; loss of Foxp3 function in humans and rodents results in devastating autoimmunity (Jordan et al., 2001; Seddiki et al., 2006; Toker et al., 2013). A vast majority of Foxp3 + Tregs are generated during T cell development in the thymus (Pacholczyk and Kern, 2008).

Type 1 diabetes is characterized by immune-mediated destruction of pancreatic β-cells, causing lifelong dependency on exogenous insulin. Autoimmunity is an outcome of an imbalance between anti-inflammatory/pro-inflammatory immune cell ratios. These ratios decide the fate of the progression of the disease which has been well established in human T1D (Ferraro et al., 2011).

Some authors speculate that Treg cells in diabetic patients turn off their FOXP3 expression once they have migrated to the pancreas (Viisanen et al., 2019). This leads to a defective control of Th17 cell population, which expands and causes the destruction of pancreatic β-cells by the release of IL-17 cytokines. Ferraro and colleagues described a case of a diabetic patient who had preserved fasting C-peptide levels 9 years after disease onset. The lymphocytes from the peri-pancreatic lymph node of the reported T1D patient showed IL-17 production upon GAD65 stimulation and displayed a very limited Treg suppressive ability in polyclonal assays (Ferraro et al., 2011).

In our previous work, we correlated the Treg/Th17 and Treg/Th1 ratios with the functionality of β-cells’ insulin synthesis in a T1D mouse model (Imam et al., 2019). Plasticity of T helper cells has been well documented, and especially, Th17 cells were reported to acquire Th1 phenotypes (Annunziato et al., 2007). Some of the Th17 cells present in Crohn’s disease were able to produce both IL-17 and IFNγ, and it been suggested that some of these proinflammatory Th17 cells may act like the Th1 type as well (Annunziato et al., 2007). Previously, we evaluated the effect of elF5A inhibition on CD4 + T cells co-expressing IL-17 and IFNγ (IL-17 + IFNγ +). CD4 T cells co-expressing IL-17 and IFNγ (IL-17 + IFNγ +) are strongly associated with plasticity of Teffector toward Treg cells and have a capacity to tip the balance toward T-cell regulation (Imam et al., 2019). Our group also reported that the increase in the Treg/Teffector cell (Th17 or Th1) ratio significantly increases the total pancreatic insulin content in humanized T1D mice. These findings show that there is a strong association between the Treg/Th17 and Treg/Th1 ratios and the functionality of islet β-cells in our humanized T1D mouse model (Imam et al., 2019); however, the increase of these ratios did not reduce cytotoxic CD8 T cells in the islets. Therefore, our previous study illustrates that interventions solely targeting CD4 T cell subsets (T helper and Treg) may not be able to revert T1D, at least in our humanized T1D mouse model (Imam et al., 2019).

Next, we looked into other modulators, which regulate Treg differentiation as well as ameliorate the cytotoxic T lymphocyte (CTL) function simultaneously. Notch signaling mediates peripheral tolerance via FOXP3-dependent mechanisms (Tran et al., 2013) as well as regulates maturation, activation, and differentiation of naive CD8 T cells into CTLs (Cho et al., 2009; Kuijk et al., 2013). Inhibition of Notch signaling using anti-DLL4 has been reported during the induction phase of experimental autoimmune encephalomyelitis in C57BL/6 mice, by increasing the pool of regulatory T cells (Tregs) in the periphery and in the CNS (Bassil et al., 2011). Others have reported inhibition of Notch signaling in an NOD mice model, using alpha-secretase inhibitors or soluble DLL4-Fc which reduces the expansion of antigen-specific CTLs in pancreatic β-cells (Billiard et al., 2012, 2018). Anti-DLL4 treatment also promotes intrathymic immature dendritic cell development, which helps in the enrichment of antigen-specific Treg cells by a mechanism that requires MHCII expression on DCs and enhances glucose-stimulated insulin secretion shown to improve islet function (Billiard et al., 2012, 2018).

Interventions using elF5A inhibition with GC7 of CD4 T cell subsets (T helper and Treg) resulted in amelioration of T1D but was not able to revert T1D, at least in our humanized T1D mouse model until interventions like anti-DLL4 restrained autoreactive CTLs in the islet microenvironment. Further, in this paper we report the simultaneous blockade of Notch and elF5A signaling using anti-DLL4 and GC7 which enriches the antigen-specific Treg cell subset collectively, and depletes the CD8 T cell subset in the pancreatic microenvironment.

## Materials and Methods

### Mice

C57BL/6-BTBR congenic mice carrying RIP-hGAD65-deficient murine MHC-class II molecules (mII-) were generated with the HLA-DQA1^∗^0301/DQB1^∗^0302 (DQ8) transgenic line that expresses HLA-DQ8 class II in the absence of endogenous murine MHC class II molecules in APCs and hGAD65 in pancreatic beta-cells. Transgenes were verified by fluorescence-activated cell sorter (FACS) and PCR. Congenic-transgenic mice were selectively in-crossed based on high fasting blood glucose for >30 generations to produce a mouse that develops diabetes spontaneously (Imam and Jaume, 2020; Imam et al., 2020, 2021). The University of Toledo Animal Research committee approved all animal breeding and research protocols.

### Genotyping of DQ8 MHC II Haplotype by Fluorescence-Activated Cell Sorter

Peripheral blood mononuclear cells (PBMCs) were isolated from the tail vein of T1D mice, and the FACS was used for sorting a heterogeneous mixture of PBMCs. PBMC pellets were suspended in staining buffer containing anti-HLA-DQ8 (leu-10) conjugated with FITC and anti-murine MHC class II conjugated with phycoerythrin (PE). Homozygosity HLA-DQ8 was determined simultaneously for the presence of DQ8 expression and absence of mII antigens using FACS Canto (BD Biosciences) and analyzed by FLOWJO software (Tree Star Inc.) (Imam and Jaume, 2020; Imam et al., 2020).

### Genotyping of RIP and hGAD65 Transgene by PCR

Genomic DNA was isolated from the tail tip of T1D mice using a ChargeSwitch^*TM*^ gDNA Mini Tissue Kit (Thermo Fisher Scientific) for genotyping of homologous RIP and hGAD65 genes. The RIP-hGAD65 gene was amplified using PCR 5′ primer from the 5′ untranslated sequences of RIP (AAGTGACCAGCTACAGTCGG) and a 3′ primer from the coding region of the human GAD65 gene (AGCA GGTCTGTTGCATGGAG). The amplified product (400 bp) was resolved on a 1.5% agarose gel (Imam and Jaume, 2020; Imam et al., 2020).

### Administration of Anti-DLL4

DLL4 (delta-like 4) Armenian hamster anti-mouse, functional grade, clone: HMD4-1 (Cat# 16594885, Invitrogen) and control Armenian hamster isotype control IgG (Cat# 16488885, Invitrogen) were intraperitoneally administered at a dose of 10 mg/kg body wt. once a week for 2 weeks. Anti-DLL4 and control IgG-treated mice were sacrificed after 30 days of the second treatment.

### Fasting Blood Glucose, Glucose Tolerance Test (GTT), Glucose-Stimulated Insulin Secretion (GSIS), and Serum Insulin

Fasting blood glucoses were measured weekly by tail vein nicking. Mice were also subjected to the glucose tolerance test (GTT) where animals fasted for 8–10 h before being administered an intraperitoneal injection of glucose (2 g/kg body weight). Blood glucose concentrations were measured at 0, 20, 30, 60, 90, 120, 150, 180, and 210 min using the tail vein nicking technique, and blood glucose was measured with an Ascensia Breeze Glucometer (Bayer). Simultaneously, at glucose challenge, serum insulin concentrations (GSIS) were measured at 0, 2, 10, and 30 min. The insulin concentration was measured by a mouse ultrasensitive insulin ELISA kit (Crystal Chem, Inc.) (Imam et al., 2019, 2020, 2021; Imam and Jaume, 2020).

### Anti-GAD65, IA2, and Insulin Autoantibody Measurement

Anti-GAD65, anti-IA2, and anti-insulin autoantibodies were measured in mice serum using an Anti-GAD65 ELISA kit (Kronus, Star, ID) according to the manufacturer’s instructions (Imam et al., 2019, 2020, 2021; Imam and Jaume, 2020). Mice anti-insulin antibodies were measured using a mouse insulin autoantibody (IAA) ELISA kit (Abbexa, catalog# abx053161, Abbexa LLC, Houston, TX, United States; with a positive predictive value range between 0.16 and 10 ng/ml). Anti-IA-2 autoantibodies were also measured using a human IA-2 autoantibody (IA-2Ab) ELISA kit (Kronus, Star, ID) following the manufacturers’ manual.

### Procurement of Organs

After 30 days of the second dose, anti-DLL4/IgG control mice were sacrificed. Sera were saved for autoantibodies and insulin assays. Pancreases were saved for histochemistry and islet scoring. Pancreases, spleens, and peri-pancreatic lymph nodes (PLN) were isolated and processed for flow cytometric analysis.

### Immune Cell Profiling

In all flow cytometry studies, the SP, PLN, and PN cells were isolated by the mechanical method to form single cell suspensions. Cell surface staining was performed by incubating 5 × 10^6^ cells with fluorochrome-conjugated antibodies against mouse CD3 (clone 145-2C11, APC, APCCy7), CD4 (clone H129.19, PECy5), CD8 (clone 53-6.7, PECy7), CD25 (clone PC61, PE), (BD Biosciences), or isotype controls for 20 min on ice, and were subsequently washed with buffer. A subset of T cells was permeabilized with cytofix/cytoperm fixation and permeabilization solution (BD Biosciences). Intracellular staining was performed with fluorochrome-conjugated antibodies against mouse IL-17 (clone 559502, PE), IFNg (clone 554413, APC), and forkhead box P3 (FOXP3) (clone MF23, Alexa Fluor 488, Alexa Fluor 647) as previously described (Imam et al., 2019, 2020, 2021; Imam and Jaume, 2020). Hoechst 33342 (10 μg/ml) staining was done to gate live cells containing 2n-4n cellular DNA. A BD FACSAria IIu/FACS Canto flow cytometer (BD Biosciences) was used to acquire the cells. The data were analyzed using FLOWJO software (BD Biosciences).

### Morphological Analysis of Pancreatic Islets and Insulitis Score-Degree Classification

Pancreases were fixed in 10% buffered formalin and embedded into paraffin. Pancreas sections (2-μm thickness) were deparaffinized and stained with hematoxylin and eosin. Hematoxylin/eosin (H&E) slides were analyzed by an optical microscope for histological identification, localization of lymphocytic infiltration, and for classification of islets with disturbed architecture as previously described (Colvin et al., 2013; Imam et al., 2019, 2020, 2021; Imam and Jaume, 2020). Insulitis scores were determined using the grading scheme: grade 1: no islet-associated mononuclear cell infiltrates; grade 2: peri-insulitis affecting <50% of the circumference of the islet without evidence of islet invasion; grade 3: peri-insulitis affecting >50% of the circumference of the islet without evidence of islet invasion; grade 4: islet invasion. An insulitis score was obtained by dividing the total score for each pancreas by the number of islets examined. Approximately 15–20 islets/pancreas were evaluated, data were represented as mean insulitis score ± SEM.

### Autoantigen Specific Proliferation of Diabetogenic T Cells

Purified CD4, CD8, and CD25 cells were isolated from T1D mice using the mice CD4 T Cell Isolation Kit (# 130-104-454), CD8a + T Cell Isolation Kit II (# 130-095-236), and CD4 + CD25 + Regulatory T Cell Isolation Kit (Cat no: 130-091-041) following standard protocol. Briefly, after sacrificing the T1D mice, pancreatic lymph nodes (PLN) were isolated and single cell suspensions were prepared. Non-CD4 T cells and non-CD8 T cells were isolated using magnetically labeled microbeads. Non-CD4 T cells were retained in the MACS column and non-touch enriched CD4 and CD8 cells were eluted from the column. Simultaneously CD25 positive cells were separated from the CD4 elute with anti-CD25-PE microbeads with more than 90% purity. Single cell suspensions of CD4, CD8, and CD25 T cells were stained with carboxyfluorescein succinimidyl ester (CFSE) to track the induced proliferation. Single cell suspensions of purified CD4, CD8, and CD25 T cells (CFSE-labeled) were co-stimulated with recombinant human GAD65 (rGAD65) protein (4 μg/ml), GC7 (100 μM), anti-DLL4 (10 μg/ml), rGAD65 + GC7, rGAD65 + GC7 + anti-DLL4, or CD3 + CD28 stimulated for 4 days (*n* = 7). CD4 T cells (CFSE-labeled) were further stained with fluorochrome-conjugated antibodies against mouse IL-17 (clone 559502, PE), IFNg (clone 554413, APC), and forkhead box P3 (FoxP3) (clone MF23, Alexa Fluor@488, Alexa Fluor@647) as previously described (Imam et al., 2019, 2020, 2021; Imam and Jaume, 2020). *In vitro* proliferation assays were analyzed by FLOWJO V10 Beta software using fix ratio, fix CV, and fix background from un-stimulated cells.

### General Statistical Analysis

For glucose and insulin concentrations, anti-GAD65, anti-IA2, anti-insulin, flow cytometric data, and GTT analyses were done separately for male and female mice with a two-way ANOVA for main effects of group interactions. The significant main effects were further tested to locate the difference in means by a least significant difference test (for differences among time points in GTT for example). Data were statistically analyzed by the SAS MIXED procedure (version 9.3, SAS Institute, Inc.). The statistical significance threshold was set at *P* ≤ 0.05. Probabilities between *P* > 0.05 and *P* ≤ 0.10 were regarded as approaching significance. Data are presented as the mean ± SEM.

## Results

### Spontaneous Type 1 Diabetes Development in Humanized Transgenic Mice

We generated a spontaneous humanized mouse model of T1D. GAD65-specific immune cells attack and destroy the pancreatic beta-cells which ultimately causes type 1 diabetes. All known stages of human T1D are recapitulated in our humanized mouse model. Moreover, our mice model develops all the classic complications of diabetes like retinopathy, nephropathy, and neuropathy (Imam et al., 2020).

First, we developed congenic C57BL6 and BTBR mice with compromised beta-cell neogenesis/regeneration (Imam et al., 2020, 2021). The congenic mice were made null for murine MHC-class II molecules (mII-) and were transduced with human HLA-DQ8 and GAD65 genes separately. After selective breeding of the congenic colony of mice carrying double-transgenes (DQ8-hGAD65 + / +), we were able to produce experimental animals with compromised beta-cell function (Imam and Jaume, 2020; Imam et al., 2020, 2021). For quality control, homozygosity of DQ8 and hGAD65 was continuously monitored using FACS and PCR (Imam et al., 2019, 2020, 2021; Imam and Jaume, 2020). Congenic mice with two human transgenes (HLA-DQ8 and GAD65) were subsequently crossed based on highest fasting blood glucose. After selective breeding of more than 30 generations, a founder animal was developed with spontaneous diabetes with a blood glucose of 350 mg/dl while other littermates had normal blood glucose (Imam et al., 2019, 2020, 2021; Imam and Jaume, 2020). Spontaneous T1D mice develop diabetes spontaneously as early as the 4th week of age. Most importantly, both sexes develop T1D in our spontaneous mouse model almost equally (as humans do).

#### Administration of Anti-DLL4

**FIGURE 1:**
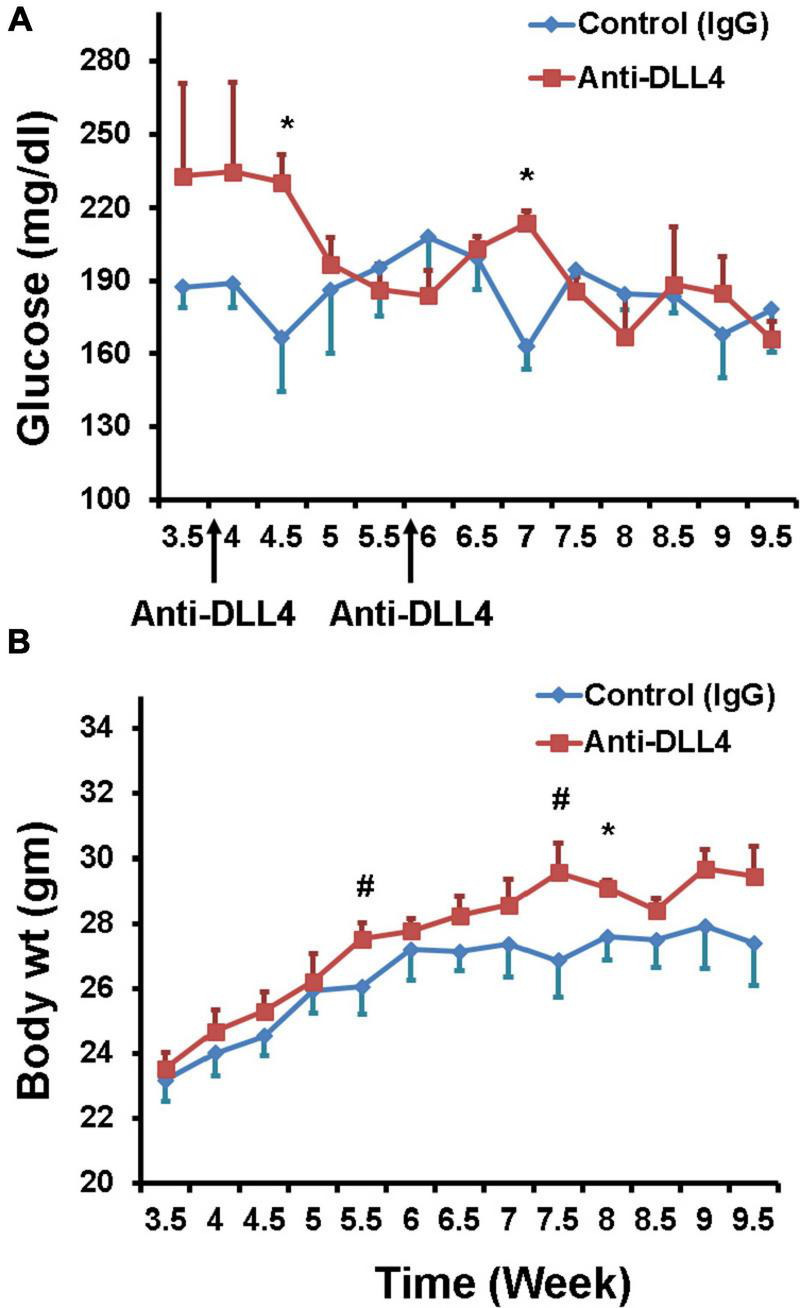
Glycemic effects of anti-DLL4. **(A)** Weekly fasting blood glucose in anti-DLL4 and control IgG treated groups (*n* = 4 per group). **(B)** Weekly body weight in anti-DLL4 and control IgG treated groups (*n* = 4 per group). Statistical significance was determined at *P* < 0.05(*), means with different superscript (#) have an approaching significant difference (*P* = 0.06 to *P* < 0.1) between the groups.

Two intra-peritoneal injections of anti-DLL4 were given at the dose rate of 10 mg/kg body weight once in 2 weeks to our recently developed T1D mouse model (Imam et al., 2019, 2020, 2021; Imam and Jaume, 2020). Weekly blood glucose data revealed that blood glucose was reduced significantly in the anti-DLL4-treated group after the first and second treatment. Reduction in weekly glucose was maintained until the 10th week with a slight fluctuation (Figure 1A), while there were hardly any effects on body weight (Figure 1B), although the anti-DLL4-treated group had a comparatively higher body weight.

#### Administration of Anti-DLL4 Significantly Reduces CD8 T Cells and Enriches the Treg Population

**FIGURE 2:**
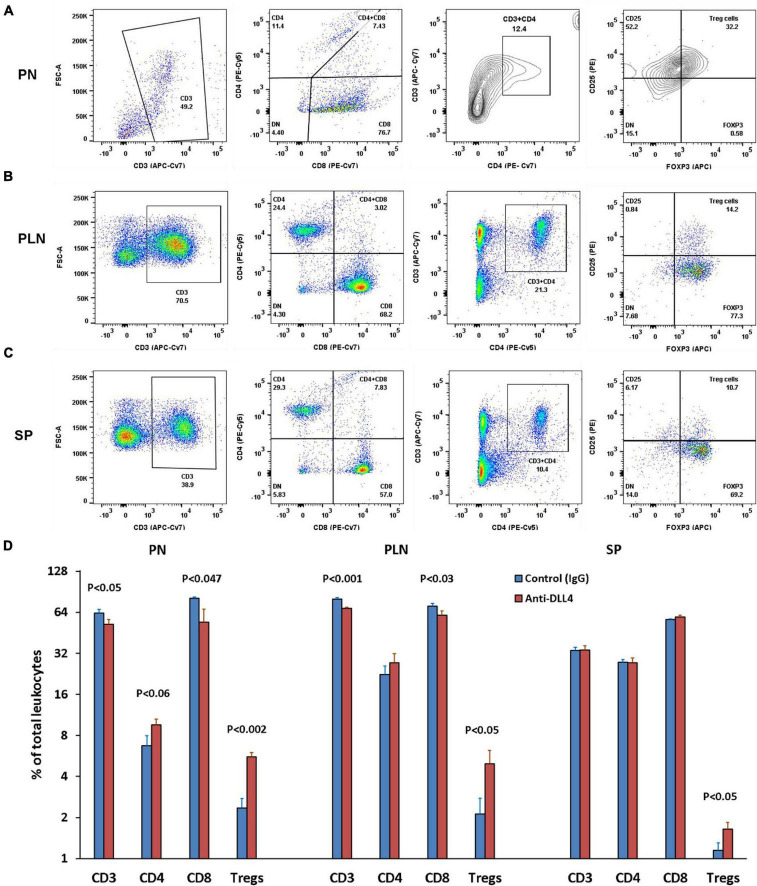
Anti-DLL4 significantly reduces CD8 T cells and enriches the Treg population. Representative flow-cytometry (dot plots) of three anatomical sites. **(A)** Pancreas (PN) **(B)** pancreatic lymph nodes (PLN) and **(C)** spleen (SP) of mice. Single cell suspensions were stained with fluorochrome-conjugated antibodies. First CD3 T cells were gated from the peripheral blood mononuclear cells (PBMCs) and subsequently gated for CD4, CD8 and (CD4 + CD25 + FOXP3) Treg cells from all anatomical sites (*n* = 4 per group). Data shown in histograms for CD3, CD4, CD8 and Treg cells were found in anti-DLL4 and Control IgG treated mice **(D)**. Most remarkably, at PN and PLN, CD3 and CD8 T cells were significantly reduced in anti-DLL4 treated group while Treg enrichment was recorded in PN, PLN, and SP of anti-DLL4 treated group.

Our data showed that inhibition of Notch signaling using anti-DLL4 significantly reduced the CD3 subset in the pancreatic microenvironment (PN and PLN). Reduction of CD3s was followed by reduction in CD8 T cells in the same organs (PN and PLN). We investigated the reduction in CD3s, and found that the reduction was actually of CD8s, which led to a reciprocal increment of CD4 Treg cells. Consecutively, inhibition of Notch signaling significantly enriched the Treg population at PN (Figure 2A), PLN (Figure 2B), and SP (Figure 2C). Most interestingly, we observed that depletion of CD8 was at the expense of enrichment of the Tregs phenotype (CD3 + CD4 + CD25 + FOXP3 +) (Figure 2D) and (CD3 + CD4 + CD25 +) (data not shown). We also observed an overall increase in CD25 expression in the CD4 T cell subsets (data not shown).

#### Administration of Anti-DLL4 Significantly Enriches the Thymic Treg Population

**FIGURE 3:**
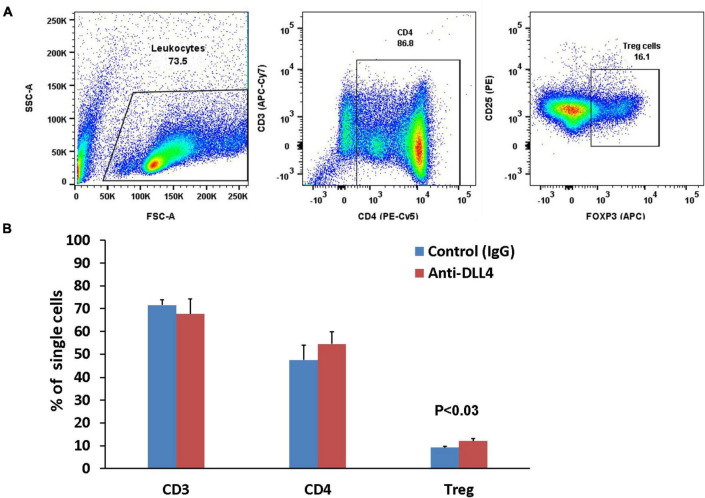
Anti-DLL4 significantly enriches the thymic Treg population. Representative flow-cytometry (dot plots) of thymus **(A)** of mice single cell suspensions were stained with fluorochrome-conjugated antibodies. Total leucocytes were gated from single cells and subsequently gated for CD3, CD4 and Treg cells (*n* = 4 per group). Data shown in histograms for CD3, CD4 and Treg cells were seen in anti-DLL4 and control IgG treated mice **(B)**. Most remarkably, significant Treg enrichment was observed in anti-DLL4 treated group.

The majority of conventional Treg cells are generated in the thymus. Thymic Tregs are permanent Tregs and inhibition of Notch signaling using anti-DLL4 significantly enriched the thymic Treg populations followed by enrichment of the thymic CD4 T cell population (Figures 3A,B). Although anti-DLL4 treatment enriched the CD4 positive population, it could not obtain the level of significance (*P* < 0.22).

#### Administration of Anti-DLL4 Significantly Protects the Islet Architecture and Improves Glucose Tolerance

**FIGURE 4:**
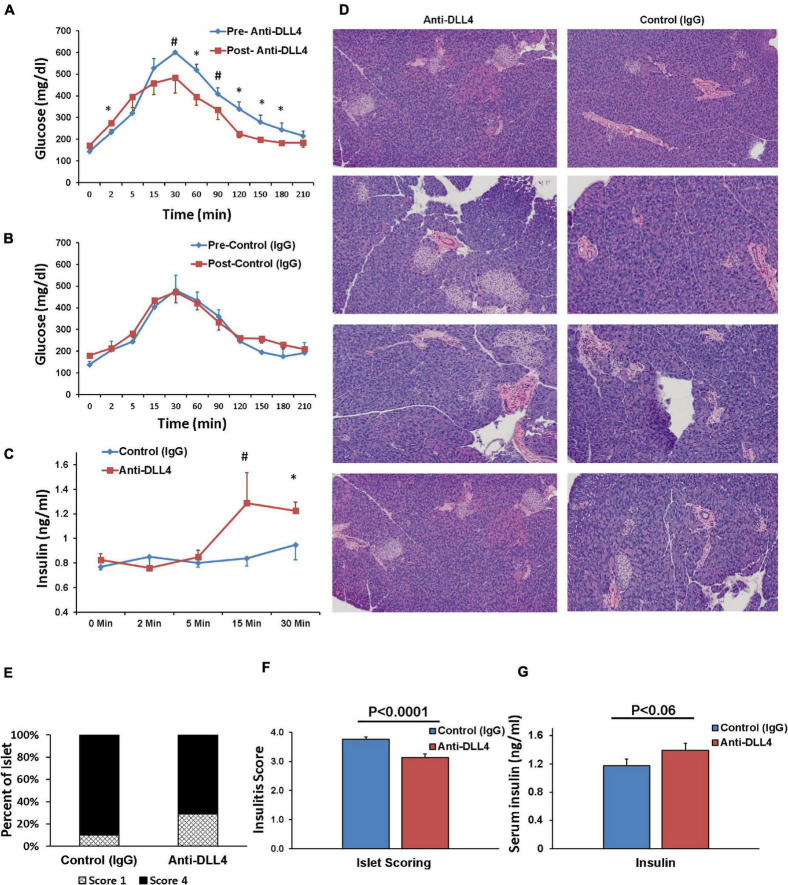
Anti-DLL4 improves glucose tolerance, protects islet architecture and ultimately improves insulin secretion. Glucose tolerance test (GTT) readings pre-treatment and post 1 month treatment of **(A)** anti-DLL4 and **(B)** control IgG treated mice (*n* = 4 mice per group). Simultaneously, **(C)** glucose stimulated plasma insulin synthesis (GSIS) were measured at 0, 2, 10, and 30 min post glucose challenge in both anti-DLL4 and control IgG treated mice (4 mice per group). Statistical significance was determined at *P* < 0.05 (*), means with different superscript (#) have an approaching statistical difference (*P* = 0.06 to *P* < 0.1) between the group. **(D)** Pancreases were isolated from mice and fixed in 10% buffered formalin. Pancreases sections (2-μm thickness) were stained with hematoxylin and eosin for morphological analysis, insulitis score-degree for histological identification, localization of lymphocytic infiltration and for classification of islets with disrupted architecture. Anti-DLL4 treated mice rescue the pancreatic islets **(E,F)** in humanized T1D mice. In totality, anti-DLL4 treatment improves the plasma insulin secretion (*P* < 0.06) as compared to control IgG treated group **(G)**.

We compared the effect of anti-DLL4 treatment on glucose tolerance (GTT) pre and post anti-DLL4 administration. Intraperitoneal administration of anti-DLL4 increased the glucose tolerance at 30, 60, 90, 120, 150, and 180 min after glucose challenge (2 g/kg body wt.). The effect of anti-DLL4 treatment was significant (*P* ≤ 0.05) at 60, 120, 150, and 180 min while the effect was approaching significant (*P* ≤ 0.06–0.1) at 30 and 90 min as compared to pre vs. post anti-DLL4 treatment (Figure 4A), whereas, in control (IgG) pre- and post-treatment, no significant differences were recorded (Figure 4B). Glucose-stimulated insulin secretion (GSIS) also followed the pattern of GTT; insulin secretion increased after 5 min of glucose challenge (GTT), and increased secretion was recorded up to 30 min post glucose challenge in the anti-DLL4-treated group (Figure 4C). We further investigated the islet architecture in anti-DLL4 and control (IgG)-treated pancreases. Anti-DLL4 treatment improved the islet architecture as well as increased the number of islets per pancreas (Figure 4D). The islet infiltration was scored on the criteria given in the methods; anti-DLL4/control (IgG)-treated mice showed measurable insulitis. The insulitis scores were significantly reduced as compared to their control (IgG)-treated counterparts (*P* ≤ 0.0001, Figures 4E,F). At the end of experiment, in total, inhibition of Notch signaling altered the pathophysiology of T1D in the humanized mouse model by improving serum insulin secretion (*P* < 0.06) as compared to the control IgG-treated group (Figure 4G).

#### Administration of Anti-DLL4 Reduces Antigen-Specific Autoantibodies

**FIGURE 5:**
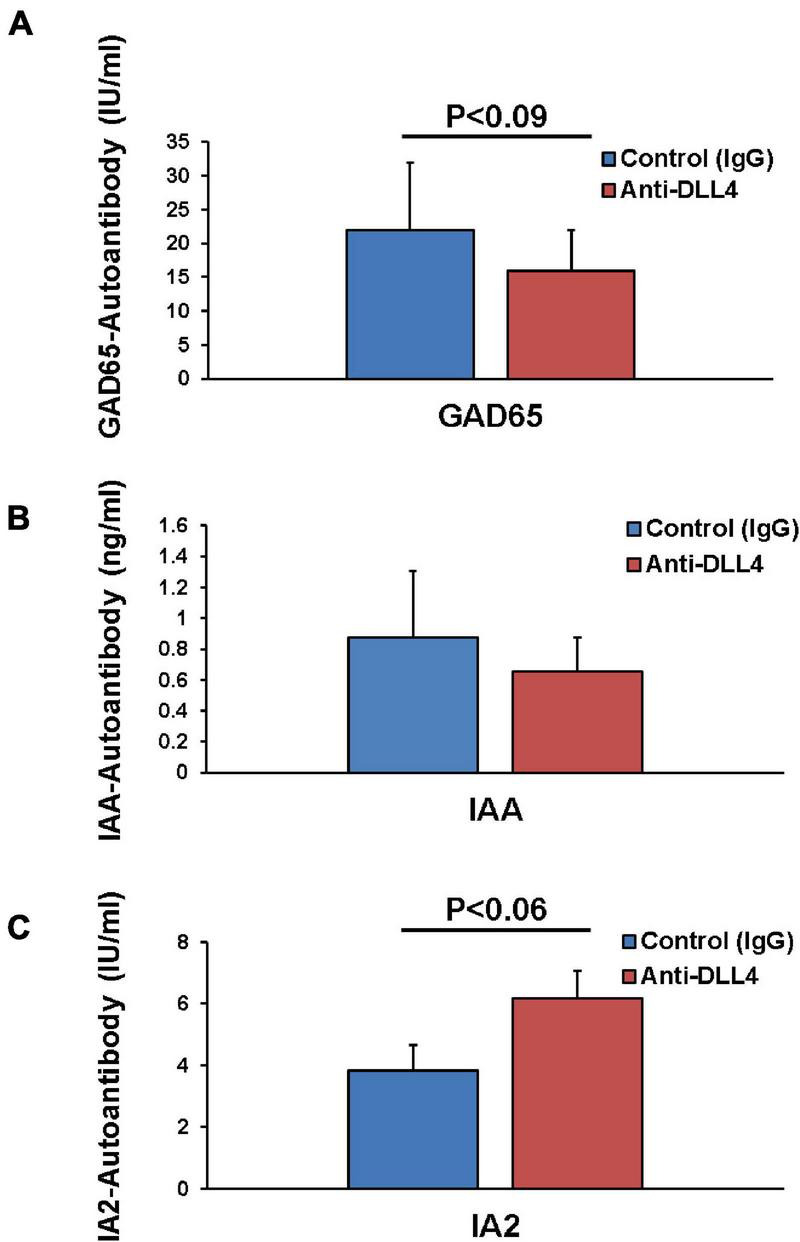
Anti-DLL4 reduces antigen specific autoantibodies. Post sacrifice of mice, sera were subjected for autoantibody analysis. Anti-GAD65 antibodies in serum were measured in anti-DLL4 and control IgG treated groups (*n* = 4 per group) **(A)**. Anti-insulin antibodies were also measured using mouse insulin autoantibody (IAA) ELISA in anti-DLL4 and control IgG treated groups **(B)** with a positive predictive value range of 0.16–10 ng/ml (*n* = 4 per group). Anti-IA-2 autoantibodies were also measured (*n* = 4 per group) using human IA-2 autoantibody (IA-2Ab) ELISA in anti-DLL4 and control IgG treated groups **(C)**.

We further investigated the effect of anti-DLL4 treatment on autoantibodies by measuring the serum GAD65, IAA, and IA2 antibodies in both treated and control groups. Administration of anti-DLL4 reduced the GAD65 (*P* ≤ 0.09) (Figure 5A) and insulin autoantibodies (IAA) (Figure 5B), while anti-DLL4 treatment increased the IA2 (*P* ≤ 0.09) autoantibodies (Figure 5C).

#### *In vitro* Expression of CD25 and FOXP3 in Tregs After Co-stimulation

**FIGURE 6:**
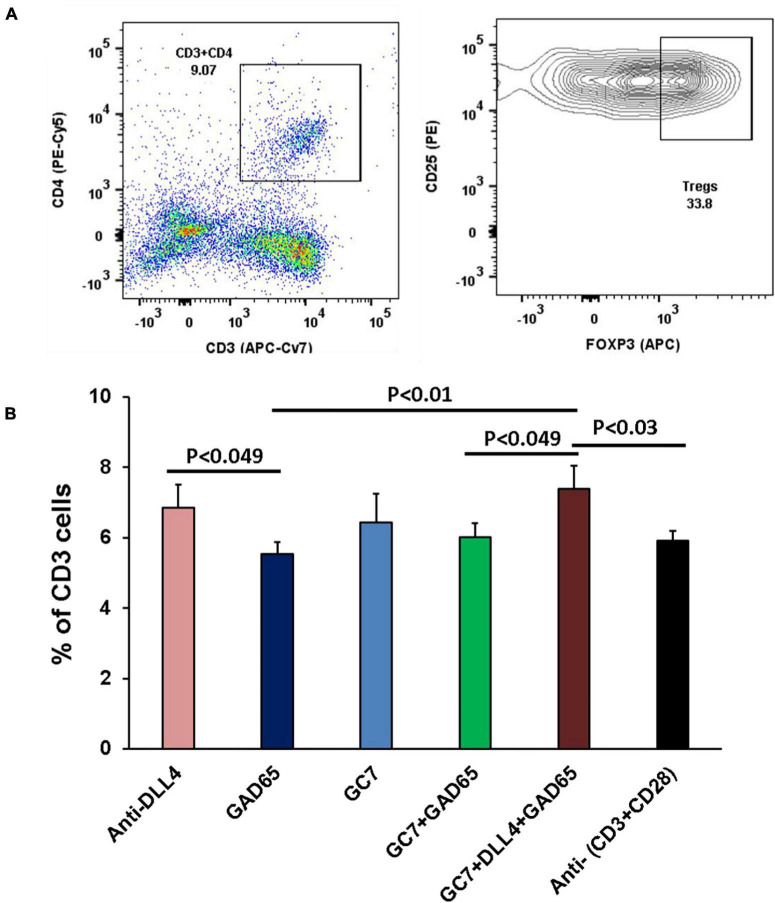
*In vitro* expression of CD25 and FOXP3 in Tregs after co-stimulation. Representative flow-cytometry (dot plots and contour plots) of *in vitro* stimulated T cells, isolated from PLN of T1D mice **(A)**. Single-cell suspensions were stained with fluorochrome-conjugated antibodies. CD3 T cells were gated for CD4 and subsequently gated for Treg cells (CD25 + FOXP3). Each sample was co-cultured in triplicate (*n* = 8 animal per group). *In vitro* stimulation with anti-DLL4, GC7, GC7 + rhGAD65, anti-DLL4 + GC7 + rhGAD65 significantly enriched Treg population **(B)** by increasing the expression of CD25 and FOXP3 on CD4 T cells.

To determine whether the modulatory effect of anti-DLL4 and GC7 is antigen-specific, we compared it with the conventional T-cell activation method using anti-(CD3 + CD28) treatment. The enrichment of the Treg population was investigated upon treatment with GC7 and/or anti-DLL4, in the presence of GAD65 autoantigen in an autoantigen-specific manner or conventionally by the use of anti-(CD3 + CD28). *In vitro* stimulation with anti-DLL4, GC7, GC7 + rhGAD65, or anti-DLL4 + GC7 + rhGAD65 specifically and significantly enriched the Treg (Figures 6A,B) population by increasing the expression of CD25 and FOXP3 (Figure 6A) on CD4 T cells.

#### Replicative Index of T Cells After *in vitro* Co-stimulation

**FIGURE 7:**
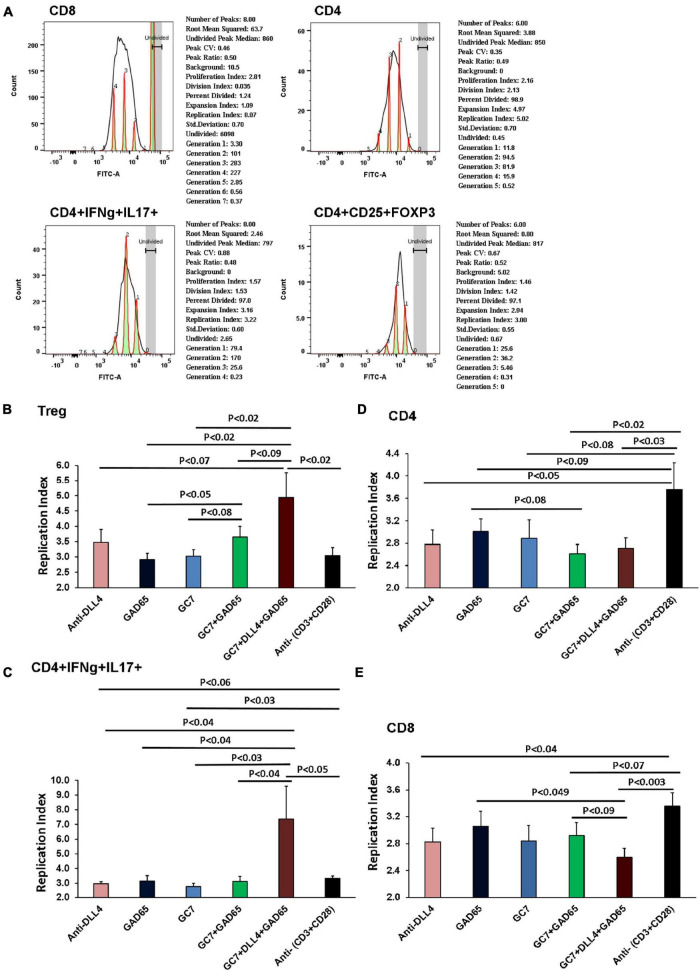
Replicative index of T cells after *In vitro* co-stimulation. Purified CD4, CD8 and CD25 were isolated from T1D mice PLNs using mice CD4, CD8 and Regulatory T Cell Isolation Kit (# 130-104-454). All T cell groups were incubated in CellTrace (CFSE-FITC, *x*-axis). Each sample was co-cultured in triplicate (*n* = 8 animal per group). Histograms show replication index as analyzed by using FLOW JO_V10 proliferation assay software **(A)**. Graph summarizes data of *in vitro* proliferation assay from above flow data. As shown, the proliferative capacity of T cells was determined by analyzing the replication of different cell types under different co-culture conditions. Replicative index of CD4, CD8, CD4 + IFNg + IL17 and CD4 + CD25 + FOXP3 cells were under *in vitro* co-stimulation conditions. Replicative index of Treg cells corresponded to the *in vitro* enrichment of Treg in anti-DLL4, GC7, GC7 + rhGAD65 and anti-DLL4 + GC7 + rhGAD65 treated groups **(B)**. We further investigated population of CD4 T cells showing plasticity toward Treg cells. Proliferative index of CD4 + IFNg + IL17 positive T cells was further analyzed using CFSE (FITC labeled) to track the individual proliferative cycle. Peak in the replicative index of CD4 + IFNg + IL17 positive T cells in anti-DLL4 + GC7 + rhGAD65 treated group was significantly higher **(C)**. Co-stimulation with anti-DLL4, GC7 + rhGAD65, anti-DLL4 + GC7 + rhGAD6 significantly reduced the CD4 count as compared to conventional stimulation with anti-(CD3 + CD28) **(D)**. Most interestingly, we also observed that co-stimulation with anti-DLL4 + GC7 + rhGAD65 significantly reduced the proliferation index of CD8 T cells **(E)**.

A significant Treg peak in anti-DLL4, GC7, GC7 + rhGAD65, and anti-DLL4 + GC7 + rhGAD65-treated groups was observed. The replicative index of Treg cells under *in vitro* co-stimulation conditions was analyzed (Figure 7A). Replicative index of Tregs cells corresponded to the *in vitro* enrichment of Tregs in anti-DLL4, GC7, GC7 + rhGAD65, and anti-DLL4 + GC7 + rhGAD65-treated groups (Figure 7B). Moreover, we investigated the population of CD4 T cells showing plasticity toward Treg cells. We sorted the CD4 + IFNg + IL-17 positive T cells and analyzed the proliferative index using CFSE (FITC-labeled) to track the individual proliferative cycles. The results revealed that there was a peak in the replicative index of CD4 + IFNg + IL-17 positive T cells in the anti-DLL4 + GC7 + rhGAD65-treated group and was significantly higher as compared to other treated groups (Figure 7C). Finally, we investigated the effect of co-stimulation (anti-DLL4, GC7, rhGAD65, GC7 + rhGAD65, anti-DLL4 + GC7 + rhGAD6) on CD4 and CD8 T cells. Our results show that co-stimulation with anti-DLL4, GC7 + rhGAD65, and anti-DLL4 + GC7 + rhGAD6 significantly reduced the CD4 count as compared to conventional stimulation with anti-(CD3 + CD28) (Figure 7D). Most interestingly, co-stimulation with anti-DLL4 + GC7 + rhGAD6 significantly reduced the proliferation index of CD8 T cells as compared to conventional stimulation with anti-(CD3 + CD28)/rhGAD65 (Figure 7E). The reduced proliferative index of CD8 T cells was not significant in the single (anti-DLL4/GC7)-treated group. This shows that synergistic inhibition of Notch signaling and elF5A using anti-DLL4 and GC7 can reduce (GAD65) antigen-specific CD8 T cell proliferation (Figure 7E).

## Discussion

The studies described underline the impact on antigen-specific regulation of Teffector cells and balance composition of the Treg cell subset in the suppression of autoreactive immunity. Moreover, these studies uncover a way of switching immune cell phenotypes from effector to regulator.

Treg cells are associated with immune tolerance and constitute 5–10% of peripheral CD4 T cells in mice and humans (Shevach, 2001). Tregs inhibit auto-aggressive/reactive effector T cells and simultaneously permit efficient defense against microbes preventing immune exacerbation and autoreactivity, which is known as the split effect. The split effect of Tregs implies that Treg activity is controlled in an antigen-specific manner. The specificity of Tregs is achieved by (i) formation of an antigen-specific Treg repertoire during their development in the thymus, and by (ii) the activation of the peripheral-tolerance by the Treg system. In the case of autoimmunity, autoantigen reactive Treg-mediated suppression operates in an antigen-specific manner that requires engagement of TCR-antigen-MHC-II to achieve significant suppressive effect on peripheral Teffector cells (Levine et al., 2014; Kieback et al., 2016). Tregs, once activated in an antigen-specific manner via their TCR, can suppress other antigen-specific Teffector cells in a bystander manner as well (Bacher et al., 2016).

Low/unfit Treg cells in T1D patients participate in the development of T1D as compared to healthy controls (Kukreja and Maclaren, 2002; Ferraro et al., 2011), and enrichment of Treg cells is an important step to fix the Treg/Teffector imbalance for suppressing autoimmunity (Darrasse-Jèze et al., 2009). Treatment with the anti-DLL4 antibody in spontaneous humanized T1D mice helps in amplifying antigen-specific Treg cell proliferation in the thymus, peri-pancreatic lymph nodes, pancreases, and spleen, which consecutively enriches peripheral GAD65-antigen-specific Treg cell population as reported previously in NOD mice (Billiard et al., 2012, 2018). Administration of anti-DLL4 Ab in T1D mice controls hyperglycemia over time and improves the glucose tolerance test (GTT). Furthermore, we show here that Notch inhibition could rescue pancreatic islets and confer protection to islet integrity in spontaneous humanized T1D mice and as an overall effect, increase insulin secretion. Normally in healthy controls, hyperglycemic stimuli triggers biphasic insulin secretion *in vivo* in humans/mice (Curry et al., 1968) and *in vitro* in perfused islets (Curry et al., 1968; Sun et al., 2008). The first phase of insulin lasts for 2–4 min while the second phase lasts up to 60–120 min. We challenged the anti-DLL4 and control IgG-treated groups with IP glucose and measured the glucose-stimulated insulin secretion (GSIS). Anti-DLL4 treatment significantly improved the second phase of stimulated insulin release. In this study, the first phase of insulin release was taken as the sum of the increments in serum insulin over the initial 10 min, and the second phase of insulin release was taken as insulin increments for up to 30 min. In diabetic patients, both phases of insulin release are reduced: the first phase is reduced by ≥19% whereas the second phase is reduced by ≥12% (Gerich, 2002) and a similar observation was seen in our IgG control group. Anti-DLL4 treatment has a similar effect to that of islet transplant in human T1D patients monitored using non-insulin-modified frequently sampled intravenous glucose tolerance (NIM-FSIGT) using euglycemic clamps, where, the second phase of insulin secretion post-transplantation was markedly increased to 83% as compared to the first phase (15%) (Vethakkan et al., 2010). A similar increment in the second phase of insulin release was noted (Figure 4C) in our study.

IA2 antibodies have been shown to be a better marker of glycemic control and of a lower insulin requirement, indicating residual beta-cell function. Therefore, we can say that Notch inhibition with anti-DLL4 improves the IA2 autoantibodies production (Figure 5C) which in turn results in a better residual beta-cell function. It has been also suggested that IA2 antibodies are closely associated with insulin secretion (Bonfanti et al., 1998; Savola et al., 1998). It may be possible that IA2 antibody production is a late occurring phenomenon. IA2 antibody production may be majorly associated with beta-cell damage and insulin release, but not necessarily with beta-cell insulin secretion from disintegrating beta-cells (Borg et al., 2001). Residual beta-cells may be giving rise to stimulated C-peptide from beta-cells that may serve as an autoantigen stimulus for further IA2 autoantibody production (Sorensen et al., 2012).

Next, we investigated the mechanism behind the Treg enrichment post anti-DLL4 antibody treatment. Most interestingly, we observed that enrichment of CD4 and the Treg phenotype (CD3 + CD4 + CD25 + FOXP3 +) (Figure 2D) and (CD3 + CD4 + CD25 +) (data not shown) happened at the expense of CD8 cells. The enrichment of Treg cells was mediated through an increase in the replicative index of Treg cells (Figure 7B). We also showed that treatment with anti-DLL4 has a complementary effect with GC7. Synergistic inhibition of Notch and elF5A signaling enriches Treg cells, which may be a downstream effect mediated through a two-fold increased replicative index of CD4 + IFNg + IL-17 + cells (Figure 7C) followed by an increased replicative index of Treg cells with similar intensity (Figure 7B).

The mechanisms by which blockage of Notch signaling ameliorates autoimmunity are not fully understood yet. This may be mediated through impaired T helper (Th1 or Th17) immune responses (Bassil et al., 2011) or impaired/reduced antigen-specific CD4 + /CD8 + T cells to the targeted organ (Cho et al., 2009; Reynolds et al., 2011), and/or promotion of regulatory T cell development (Bassil et al., 2011) at the expense of CD8 depletion (Figure 2D).

It has been hypothesized that Notch signaling upregulates the APC-mediated T helper cell responses (Amsen et al., 2004, 2009; Skokos and Nussenzweig, 2007; Sun et al., 2008), and engagement of Delta-like Notch ligands favors their development (Auderset et al., 2012), whereas our data revealed that blocking Notch signaling using anti-DLL4 increases the CD4 T cell count and the increment is mediated through increased CD4 + CD25 + FoxP3 (Treg) count. Our data are in concordance with other reports where a glucose challenge in anti-DLL4-treated mice consequently leads to better second-phase insulin release. Our results are also in line with similar experiments where Notch signaling was inhibited with γ-secretase inhibitors, which consecutively reduced the effects of experimental autoimmune encephalomyelitis (EAE) in a mouse model (Minter et al., 2005; Jurynczyk et al., 2008). In a similar observation, it has been recorded that blocking Notch signaling suppresses the deleterious effects of multiple sclerosis in another mouse model as well (Elyaman et al., 2007; Bassil et al., 2011).

Our study revealed that treatment with anti-DLL4 helped enrich the peripheral and thymic Treg population which leads to preservation of islet architecture and improved the islet infiltration scoring in terms of healthy islet count per pancreas as well as serum insulin level. It also validated previous researchers’ findings in the sense of improved immune tolerance by delaying the rejection in an MHC-mismatched heart transplantation mouse model (Riella et al., 2011). Similarly, complete Notch blockade with either anti-DLL1 and anti-DLL4 antibodies or T cell-specific ablation of Notch signaling using DNMAML, delayed cardiac allograft rejection without co-stimulation blockade (Wood et al., 2015). It has also been noted that short-term blockage of DLL1/4 signaling was sufficient to confer CD4 + protection against T cell-mediated rejection during allogeneic bone marrow transplantation which results in Treg expansion (Zhang et al., 2011).

Notch signaling is also associated with the upregulation of the transcriptional regulator eomesodermin (Eomes) which regulates the expression of perforin and granzyme B in naive CD8 + T cells and helps differentiate T cells into cytotoxic T lymphocytes (CTLs) (Cho et al., 2009). Notch1 antisense transgene and GSI-mediated inhibition of Notch signaling attenuate CTL function by decreasing the expression of Eomes, perforin, and granzyme B in mice (Cho et al., 2009), and reduces cytotoxic T cell activity in a transplant mouse model (Riella et al., 2011). Notch signaling blockage on splenic CD8 + T cells changes cytokine secretory patterns; decreases IFNγ production, and increases the production of IL-10 (Wong et al., 2003).

Taken together, we can suggest that Notch signaling participates in regulating genes necessary for CTL cytotoxicity, differentiation, and function. Therefore, treatment with anti-DLL4 ameliorates the differentiation of CTLs and its cytotoxic function. A similar observation has been recorded in our current study where anti-DLL4 alone or in combination with GC7 significantly reduced the CD8 T cell population in PN and PLN *in vivo* (Figure 2D) as well as *in vitro* by reducing the replicating index of antigen-specific CD8 T cells (Figure 7E).

Inhibition of Notch signaling prevents allograft rejection in a lung transplant mouse model by enhancing Treg survival, proliferation, and suppressive functions (Magee et al., 2019). It has been also demonstrated that expansion of Tregs was attributable to decreased apoptosis of peripheral Tregs as well as increased Treg proliferation (Magee et al., 2019). In our current study, an *in vitro* co-stimulation experiment revealed that synergistic stimulation with GC7 + anti-DLL4 enriches the antigen-specific Treg population by increasing the expression of CD25 and FOXP3 (Figure 6B) and decreasing the proliferative index of CD8 (Figure 7E). We suggest it may be because of antigen-specific IL-17 + IFNγ + producing T-helper cell plasticity within the T-helper subset, as reported previously by our group and others (Zhou et al., 2009; Imam et al., 2019).

**FIGURE 8:**
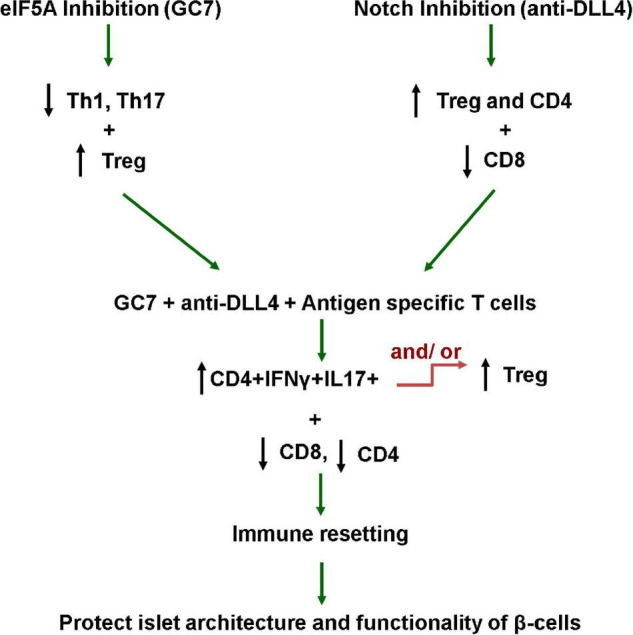
Working hypothesis. This study explains that elF5A and Notch signaling inhibition favors the reversal of the pro-inflammatory milieu, enriching the Treg population in the pancreatic microenvironment (PLN and PN), and restraining the antigen-specific CD8-mediated destruction of β-cells. This study also explains that the plasticity of IL-17 + IFNγ + producing CD4 cells toward Treg phenotype is proportional to the increased proliferative capacity of IL-17 + IFNγ + producing CD4 cells. These interventions tip the pro-inflammatory balance toward regulation and protect/rescue T1D mouse islet β-cells from autoimmune destruction.

Autoantigens presented by antigen-presenting cells lead to differentiation of naive CD4 + T cells into different subsets of T helper (Th) cells (Th1, Th2, Th17, and iTreg cells), and these differentiations are cytokines milieu-dependent. For example, T-bet is required for differentiation of Th1 cells, RORγt for Th17 cells, and Foxp3 for iTreg cells. Plasticity between Th1, iTreg, and Th17 cells has been reported under certain cytokine milieu conditions. iTregs can convert to IL-17-producing cells upon stimulation with IL-6 and IL-21, whereas Th17 cells may also reprogram into IFN-g-producing Th1 cells under stimulation with IL-12 (Zhou et al., 2009). Mechanisms behind the IL-17 + IFNγ + -producing CD4 cells’ plasticity have been documented but not defined. This is first time a study explains the plasticity of IL-17 + IFNγ + -producing CD4 cells toward Treg as proportional to the increased proliferative efficacy of IL-17 + IFNγ + -producing CD4 cells (Figure 8). Therefore, antigen-specific-mediated co-stimulation with GC7 + anti-DLL4 induces plasticity in T helper subsets toward Tregs as it is well established that Th1 and Th17 cells are microenvironment cytokine milieu-dependent (Zhou et al., 2009).

The flexibility of Treg and Th17 cell differentiation provides us with a model system where the plasticity and unstable phenotypes of Tregs, Th1, Th17, and Th17 + IFNγ + cells will have important biological implications for designing therapeutic regimens to control autoimmunity.

## Data Availability Statement

The original contributions presented in the study are included in the article/supplementary material, further inquiries can be directed to the corresponding authors.

## Ethics Statement

The animal study was reviewed and approved by the Institutional Animal Care and Use Committee (IACUC), University of Toledo.

## Author Contributions

SI: conception and designing of the study, analysis and interpretation of data, and drafting the manuscript and final approval for submission. JJ: conception and designing of the study and final approval of the version for submission. PD: designing and executing *in vitro* studies and reviewing the manuscript critically. SA: analysis and drafting the manuscript. ZZ: pathological scoring and scoring of islets. HS and TK: data analysis and helping in experimentation. SF, IH, AN, AH, and RS: *in vitro* experimentation. NS: data collection, analysis, and drafting the manuscript. All authors contributed to the article and approved the submitted version.

## Conflict of Interest

The authors declare that the research was conducted in the absence of any commercial or financial relationships that could be construed as a potential conflict of interest.

## Publisher’s Note

All claims expressed in this article are solely those of the authors and do not necessarily represent those of their affiliated organizations, or those of the publisher, the editors and the reviewers. Any product that may be evaluated in this article, or claim that may be made by its manufacturer, is not guaranteed or endorsed by the publisher.
